# Sequential self-assembly of DNA functionalized droplets

**DOI:** 10.1038/s41467-017-00070-0

**Published:** 2017-06-16

**Authors:** Yin Zhang, Angus McMullen, Lea-Laetitia Pontani, Xiaojin He, Ruojie Sha, Nadrian C. Seeman, Jasna Brujic, Paul M. Chaikin

**Affiliations:** 1 Physics Department, Center for Soft Matter Research, New York University, 4 Washington Place, New York, New York 10003 USA; 2 Chemistry Department, New York University, 100 Washington Square East, New York, New York 10003 USA; 3 Institut des NanoSciences de Paris, UMR 7588, Centre National de la Recherche Scientifique-University Pierre et Marie Curie, 4 Place Jussieu, Paris, France

## Abstract

Complex structures and devices, both natural and manmade, are often constructed sequentially. From crystallization to embryogenesis, a nucleus or seed is formed and built upon. Sequential assembly allows for initiation, signaling, and logical programming, which are necessary for making enclosed, hierarchical structures. Although biology relies on such schemes, they have not been available in materials science. Here, we demonstrate programmed sequential self-assembly of DNA functionalized emulsions. The droplets are initially inert because the grafted DNA strands are pre-hybridized in pairs. Active strands on initiator droplets then displace one of the paired strands and thus release its complement, which in turn activates the next droplet in the sequence, akin to living polymerization. Our strategy provides time and logic control during the self-assembly process, and offers a new perspective on the synthesis of materials.

## Abstract (web summary)

Natural complex systems are often constructed by sequential assembly but this is not readily available for synthetic systems. Here, the authors program the sequential self-assembly of DNA functionalized emulsions by altering the DNA grafted strands.

## Introduction

Over the past decade, there has been growing interest in using colloids for the self-assembly of arbitrarily designed structures^1–4^, rather than the more conventional colloidal crystals fabricated over the past century^5–8^. Colloidal particles with specific interparticle recognition facilitated by their functionalization with DNA sticky ends^9–11^, lock and key interactions^12^, as well as particles of different shapes and symmetrically arranged patches^13, 14^, have led to theoretical proposals for the creation of demonstrative target structures, e.g., a colloidal version of the Empire State building^4^. Such proposals rely on self-assembly through tunable enthalpic and entropic interactions guiding the system toward thermodynamic equilibrium. In addition, introducing mobile linkers to colloids^15^, liposomes^16, 17^, and droplets^18–20^ avoids kinetic traps along the self-assembly pathways. However, the design principle that invokes sequential self or directed assembly has not yet been addressed due to the absence of an underlying technology. Sequential assembly is used industrially to make everything from integrated circuits to skyscrapers and is required in the growth of all living organisms. In many cases, it is a convenient organizational tool, in others, such as the three-dimensional enclosed structures of the Russian dolls, it is necessary to assemble the inside before the outside.

In this article, we demonstrate a technique for programmed self-assembly on the colloidal scale using the specific hybridization and easy design properties of DNA sequences^21^. Related schemes have been shown on molecular scales using DNA duplexes or DX motifs^22–24^. In our system, a family of diversely functionalized emulsion droplets… B, C, D,… are self-assembled such that C only binds to D after B binds to C. This process is controlled by using protected pairs of DNA strands on the droplets and a well-developed technology, toehold strand displacement^25–27^, as the enabling mechanism. With A as an initiator and E as a termination droplet, we spell out a droplet sequence ABCDE.

## Results

### Experimental design

**Fig. 1:**
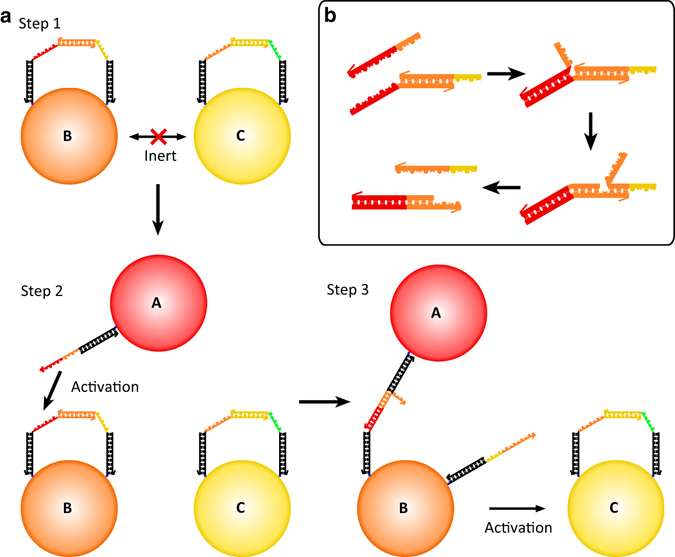
A schematic of sequential self-assembly. **a** Step 1: two droplets, B and C, with partially hybridized pairs of DNA strands are inert. Step 2: a droplet with an initiator strand, A, is introduced. Step 3: the initiator strand fully hybridizes to the protection strand on B through toehold strand displacement and frees the protected strand, leading to the activation of droplet B. The freed strand on B can then hybridize with the protection strand on C making a B–C bond and activating C for further binding. **b** Schematic of the toehold displacement reaction showing details corresponding to the activation process of (**a**)

In our design, we graft mobile DNA linkers to micro-scaled emulsion droplets, as depicted in Fig. 1a. In step 1, a set of droplets B and C have been prepared with pairs of DNA strands that are protected by their partial hybridization. In practice, each droplet has tens of thousands of such pairs. The protection renders the droplets inert—no bonds are formed between protected droplets. The complementary orange DNA sequences on B are hybridized and protected from the unpaired orange sequence on C. Similarly, the paired yellow sequences on C are protected from the unpaired yellow DNA strand on B. In step 2, an initiator droplet, A, is introduced. The DNA sequence on A has a section that is complementary to the unpaired red sequence and half of the paired orange sequence on B. Following the scheme of the toehold strand displacement mechanism (Fig. 1b), the complementary red sequences and half of the orange sequence bind by the DNA strand-branch migration. With proper sequence design, the remaining half of the originally hybridized yellow sequence is above its melting temperature and unbinds. Droplet A and B are then bound and the orange yellow DNA single strand is left unbound. If it were attached to a solid colloidal particle, it would be localized to the region near the A–B binding site. However, on an emulsion droplet, it is free to diffuse over the entire droplet surface, as shown in step 3 of Fig. 1a. Droplet B is then activated and can serve as an initiator for binding and activation of droplet C.

DNA functionalized droplets are different from similarly functionalized colloids in several ways related both to the mobility of the DNA ligands and the deformability of the droplets^28–31^. When droplets approach, a few DNA strands on one droplet can hybridize with complementary strands on the other droplet making an initial bond. However, surface diffusion allows additional DNA strands to migrate to the contact region to form additional hybridized bonds and a growing patch. The patch flattens the interface between the droplets at the cost of elastic energy (surface tension). The balance between the hybridization energy and the elastic energy gives rise to an equilibrium patch size with a given number of strands, *N*_s_. If the number of DNA strands on a droplet is less than or equal to the equilibrium *N*_s_, then the droplet should be monovalent and binds to only one other droplet. In the present case, we use a low concentration of initiator strands and a low volume fraction of droplets to ensure that we are in the monovalent regime for each binding event. The number of released strands must be less than or equal to the number bound in each previous step in the sequence.

**Fig. 2:**
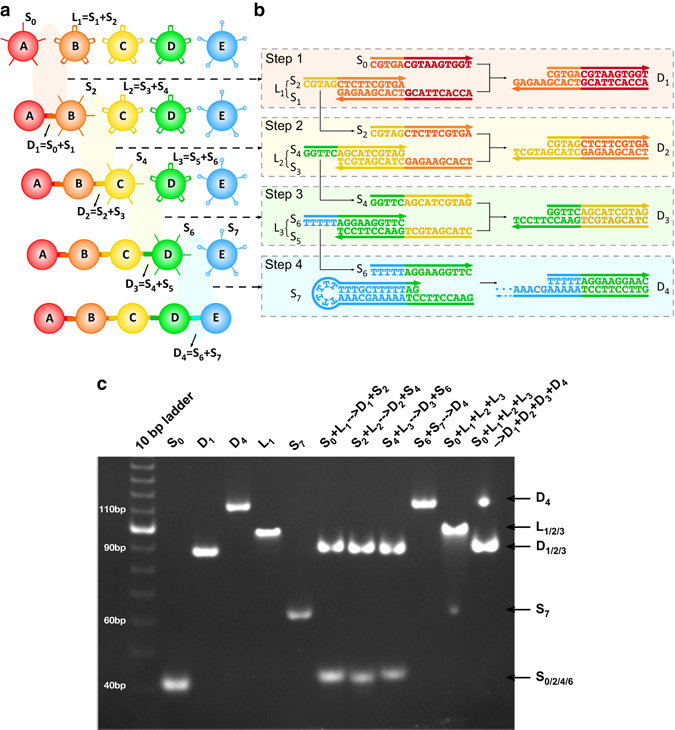
Experimental design and gel results. **a** The programmed assembly sequences of the fluorescently labeled droplets used in this study. **b** The toehold strand displacement in each step corresponds to the sequential binding of droplets and their progressive activation of droplets in **a**. **c** Native PAGE demonstrates the step-by-step toehold strand displacement reactions depicted in **b**. Column ‘10 bp ladder’ is the marker lane. Column S_0_ serves as a reference for single strands S_2_, S_4_, and S_6_ with the similar molecular weights. Similarly D_1_ is a reference for D_2_ and D_3_ (completely hybridized duplex strands); L_1_ is a reference for L_2_ and L_3_ (partially hybridized strands), while S_7_ and D_4_ mark the terminator hairpin strand and its partially hybridized duplex strands. Columns S_0_ + L_1_ → D_1_ + S_2_, S_2_ + L_2_ → D_2_ + S_4_, S_4_ + L_3_ → D_3_ + S_6_, and S_6_ + S_7_ → D_4_ show that the reactions depicted in **b** produce the desired results and leave none of the initial reactants. Column L_1_ + L_2_ + L_3_ + S_7_ shows that the partially hybridized protected strand pairs are inert. The final column shows that the reactions go to completion when the initiator is added

As a demonstration of sequential assembly, we choose a simple design—a set of droplets each labeled with a unique color. The designed sequence of events is shown in Fig. 2a. Droplet A is the initiator, while droplets B, C, D have protected pairs of DNA strands, and droplet E is a terminator with single DNA strand protected in a hairpin configuration. The sequences for the active regions of A–E droplets are shown in Fig. 2b. Droplets B, C, D, and E are prepared separately and then added to the same bath, since they are inert. When the initiator droplets A are added, we begin to see the sequential assembly of droplets binding, illustrated in Fig. 2a, and the corresponding strand displacement reactions in Fig. 2b. The system forms dimers, trimers, tetramers, and pentamers in the correct color order (and nothing longer).

### Gel electrophoresis

Our sequential assembly scheme relies on the stability of the protected strand pairs and the effectiveness of strand displacement. These properties were tested by a series of experiments on the individual strands before attachment to the emulsion droplets. (Note that each single strand illustrated in Fig. 2b is attached to a 36 nucleotide spacer strand, which can be hybridized to a complementary strand to make a duplex spacer with a biotinylated end allowing both attachment to a fluorescent streptavidin and separation of the active region from the droplet surface.) In Fig. 2c, we show a non-denaturing gel study verifying the protections and reactions between sequences depicted in Fig. 2b. Lane S_0_ indicates the band for strand S_0_ and serves as a reference for the single strands S_2_, S_4_, and S_6_ with similar molecular weight. Similarly D_1_ is a reference for bands D_2_ and D_3_ (completely hybridized duplex strands), L_1_ is a reference for L_2_ and L_3_ (partially hybridized strands), while S_7_ and D_4_ mark the terminator hairpin strand and its partially hybridized duplex strands. Lanes S_0_ + L_1_ → D_1_ + S_2_, S_2_ + L_2_ → D_2_ + S_4_, S_4_ + L_3_ → D_3_ + S_6_, and S_6_ + S_7_ → D_4_ show that the reactions depicted in Fig. 2b produce the desired results and leave none of the initial reactants. Lane L_1_ + L_2_ + L_3_ + S_7_ shows that the partially hybridized protected strand pairs do not react in the absence of the initiator. On the other hand, the final lane shows that when the initiator strand is added to the previously inert protected strands, the complete set of reactions goes to completion with only complete duplex strands and the partially hybridized termination duplex strands as the result.

### DNA functionalization

Having confirmed the success of the molecular scheme, we next functionalize the droplets to drive their polymerization. The experiments are performed with micron-sized, lipids stabilized silicone oil in water emulsion droplets (see methods). DNA loops (L_1_, L_2_, and L_3_) are pre-annealed and purified from native gels to ensure the correct stoichiometry. A ‘biotinylated phospholipid—fluorescent streptavidin—biotinylated oligonucleotide’ linking strategy is applied to functionalize the oil droplets with the corresponding DNA structures (S_0_, L_1_, L_2_, L_3_, and S_7_). In the experiment, each combination of streptavidin with different fluorescent labels is specifically associated with one type of DNA strand/loop structure to distinguish them using fluorescence confocal microscopy. From fluorescence spectroscopy measurements, we estimate that the surface coverage of streptavidin on droplets is ~ 1000 μm^−2^ (Supplementary Methods and Supplementary Fig. 3).

### Confocal imaging and data analysis

**Fig. 3:**
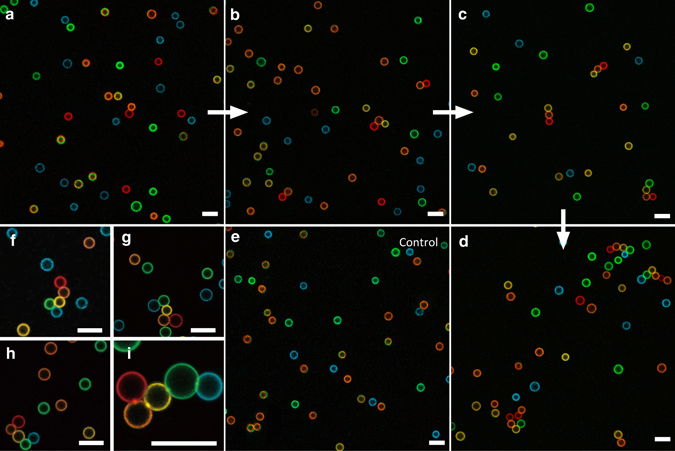
Representative confocal images of sequentially self-assembled structures. In the experiment, droplets A, B, C, D, and E were false colored red, orange, yellow, green, and blue. **a**–**d** Images taken after 10 h, 26 h, 74 h, and 101 h of incubation, respectively, showing the growth of triggered sequential self-assembly (SSA). **e** An image taken from the control experiment (no initiators) after 101-h incubation. **f**–**h** A gallery of various self-assembled pentamers. **i** A zoom-in view of a pentamer showing the patch formed at each droplets binding. All scale bars are 10 μm

**Fig. 4:**
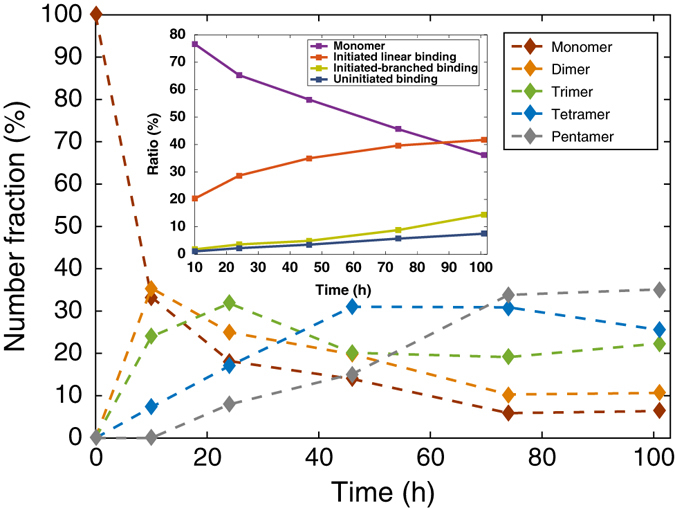
The evolution of the distribution of assembled chains of various lengths. Each data point is the ratio of the number of initiators in chains of a certain length to the total number of initiators from the analysis (see Supplementary Tables 1 and 2 for the counting results). Each curve demonstrates the change of the number fraction of the assembled structures of a certain length with time. This group of curves presents the majority of the assembled structures in the system progress from monomers to dimers, trimers, tetramers, and finally pentamers. The *inset* shows the proportions of droplets staying as unbound monomers, with initiated linear (only one predecessor and one successor) and branched (multiple predecessors or successors) bindings, as well as non-specific bindings at different time points. The droplets with non-specific bindings account for the ones in wrong sequences (e.g., A—C, B—E or D—D, etc.) or uninitiated assemblies (e.g., B—C or C—D—E, etc.)

After DNA functionalization, the droplets are mixed together at a number ratio of A:B:C:D:E = 1:2:2:2:2, and loaded into a homemade sample chamber (Supplementary Methods). The droplets cream to the top surface forming a monolayer, and diffuse thermally. The chamber was placed on a leveled surface in an incubator at 12 °C. The sample is imaged using fluorescence confocal microscopy (Leica TCS SP5 II) at different time points. Confocal images demonstrate the growth of sequential clusters with initiators (Fig. 3a–d and Supplementary Figs. 3–7) and a control without initiator (Fig. 3e). The control shows little aggregation after 101 h with ~ 87% of the droplets staying unbound. The experiments with initiators show the droplets first self-assemble into dimers, then trimers, tetramers, and finally pentamers (Fig. 3f–i). Figure 4 quantifies these results showing that, in the presence of the initiators, by 101 h the dominant population (35% of the initiators) is the programmed pentamer in the correct sequence, and 94% of the initiators have been used up. Another analysis (Fig. 4 *inset*) indicates that initiated sequential bindings formed in the system are six times more than the number of mistakes (uninitiated bindings or bindings in the wrong sequence). Among the initiated sequential bindings, 73% are in linear regime (only one predecessor and one successor) and 27% in branched regime (multiple predecessors or successors). At present, the control of monovalent binding is less than perfect. Reducing the DNA surface density on droplets will further optimize our system, leading to a higher yield of linear chains.

### Kinetics model for the sequential self-assembly

The time scale for these reactions is governed by the aggregation kinetics of monolayer DNA-coated droplets on a surface as studied in ref. ^32^. The aggregation time *τ* can be obtained from:

1 $$\tau = {\tau _{{\rm{DLA}}}} + \frac{{{\tau _{\rm{R}}}}}{{4\pi {R_{\rm{d}}}L{C_0}}},$$

where

2 $${\tau _{{\rm{DLA}}}} = \frac{{ - \ln \left( {4\pi eR_{\rm{d}}^2{C_0}} \right) - 1}}{{8\pi {D_0}{C_0}}}.$$

*τ*_R_ is the reaction time of a pair of droplets with complementary sticky-end DNA on their surfaces staying within the reaction region (thickness *L* ~ 10 nm). *C*_0_ is the number density of droplets of one particular type, ~ 3 × 10^2^ mm^−2^ for droplet A and ~ 6 × 10^2^ mm^−2^ for droplets B, C, D, and E. *R*_d_ is the average radius of the emulsion droplets, ~ 2.35 μm; *D*_0_ is the measured two dimensional diffusion coefficient of droplets, ~ 0.037 μm^2^ s^−1^. Our system is reaction limited with *τ*_DLA_ ~ 0.5 h and the rate of DNA-coated droplet binding (the second term in the right hand side of Equation 1) ~ 0.2–2 h for the initiator reaction and ~ 2–20 h for the subsequent reactions (Supplementary Note 1). For hybridization to occur, the accumulated contact time for a pair of strands to be hybridized is ~1 s. Strands diffuse on the droplet surface quickly and make contact many times before binding from one droplet to another. Thus, *τ*_R_ depends on the diffusion coefficient and density of active DNA on the droplet surface. For the initiator, the number of active strands is measured by fluorescence to be ~ 12,000, corresponding to a strand density ~ 200 per μm^2^. The kinetic model allows us to estimate the number of released strands from the adhesion patch to be ~ 1200 or 20 μm^−2^ in order to match the observed assembly time scales demonstrated in Fig. 4. To test the robustness of our system, the experiment is carried out using another batch of smaller droplets (3.4 μm) with higher diffusion coefficient (0.08 μm^2^ s^−1^). The results present a similar yield (Supplementary Tables 3 and 4) and assembly kinetics (Supplementary Fig. 8), which have also been confirmed by theoretical calculation (Supplementary Note 1).

## Discussion

We have demonstrated a technique and developed a simple system to allow for the sequential assembly of micron-scale objects. The experimental parameters, such as temperature, DNA sequences, droplet density, size, and grafting chemistry can be optimized for faster growth, error reduction or increasing/decreasing branching. For instance, we can use a longer duplex in the loop structures for added stabilization, which should minimize uninitiated binding. We could also improve our yield through magnetic or surface tethered initiators, allowing us to remove the unreacted monomers and non-specific clusters. This method can be further extended in many directions. Programmed sequential self-assembly can produce stable non-equilibrium structures similar to the directed sequential growth achieved by molecular beam epitaxy^33^. A cyclic system, IABCABCABC… where the released strand on C is the same as the initiator strand would allow for an extended structure and a true emulsion analog to living polymerization. The linear chains can be folded into two or three-dimensional structures by coating surfaces with several different species of DNA, which can later be turned on chemically or by cooling. An initiator or subsequently freed strand can bind to release more than one type of strand in the next sequence step (by using the same protection strand on two partially different sequences or protecting two strands with one). This would allow control of branching and/or the direct construction of three-dimensional objects. Conversely, two or more initiator strands could be required to bind to and activate a droplet. Lipid coatings on colloidal particles enable mobility on their surfaces^15^, and thus serve as an extension of our technique to solid particles. Logic and sequence control should greatly expand the range of structures and devices accessible to colloidal/emulsion self-assembly.

## Methods

### Droplet fabrication

In the experiment two batches of droplets with different sizes were used. They were made via different methods. The 4.7 μm droplets: 5 ml of silicone oil (viscosity = 50 cst, from Sigma Aldrich) was extruded through a membrane with a mean pore size of 1 μm using a membrane emulsification device (internal pressure type micro kit, SPG Technology Co., Ltd). The droplets were stored in a 10 mM sodium dodecyl sulfate (SDS) buffer at 4 °C after two cycles of washes. The average diameter was 4.7 μm. The 3.4 μm droplets^34^: 5 ml of DMDES was vortexed with 500 μl of deionized water for 5 min, then left on a rotator for 30 min until the mixture was homogeneous. We then mixed a solution of 6% v/v prehydrolyzed DMDES and 20% v/v ammonia (total volume was 35 ml), and vortexed for 5 min. The droplets were left to grow undisturbed for 24 h, until the droplets were monodisperse and ~1.75 μm in diameter. After growth, we dialyzed the droplet solution against a 1 mM SDS solution until all traces of ammonia were removed. We then restarted the reaction by adding 1.5 ml of prehydrolyzed DMDES and 1% v/v ammonia. The PDMS droplet seeds were left to grow undisturbed until their growth had saturated. More prehydrolyzed DMDES was added until the droplets had reached the desired diameter. These droplets were again dialyzed against a 1 mM SDS solution until all unreacted monomer and ammonia were removed, and then stored in a 10 mM SDS buffer at 4 °C until the droplets were ready to be labeled with lipids.

### Phospholipids labeling

We used different methods to functionalize the two batches of droplets with phospholipids. For the 4.7 μm droplets: egg l-α-phosphatidylcholine lipids and DSPE-PEG (2000) biotin (both from Avanti Polar Lipids) were mixed at a molar ratio of 95:5, and evaporated under filtered nitrogen. The dried phospholipids were then dissolved in dimethyl sulfoxide (Sigma Aldrich) to 100 g l^−1^, and further diluted in a 10 mM Tris-HCl, pH 8, 1 mM SDS, 0.1 mM NaN_3_ buffer to 1 g l^−1^. The lipids buffer was then added to the creamed emulsion droplets at a volume ratio of 1000 μl:100 μl. Then the mixture was diluted to 10 ml using the same buffer, and tumbled gently at room temperature overnight. The emulsion was washed three times using the TMS buffer (10 mM Tris-HCl, pH 8, 3 mM MgCl_2_, 1 mM SDS, 0.1 mM NaN_3_) and then stored at 4 °C for further use. These emulsions are stable for several months. For the 3.4 μm droplets: DSPE-PEG (2000) (1,2-distearoyl-sn-glycero-3-phosphoethanolamine-N-[amino(polyethylene glycol)-2000]) and DSPE-PEG (2000) biotin (both dissolved in chloroform) were mixed at a 20:1 molar ratio. Approximately 10 μl of this lipid mixture (10 g l^−1^ in chloroform) was added to a clean glass vial. We then evaporated off all chloroform with nitrogen, and resuspended the lipid cake in 70 μl of dimethyl sulfoxide (Sigma Aldrich). To this mixture, we added 1 ml of 10 mM sodium acetate buffer (pH 5), 1 mM SDS, 1 mM EDTA. Then, 50 μl of creamed emulsion droplets were added in this solution, and left to incubate on a rotator for 24 h. The droplets were then washed three times in a 10 mM Tris-HCl pH 8, 1 mM Tergitol TMN-6, 1 mM EDTA buffer to remove any unattached lipids. Lipid-functionalized droplets were stored at 4 °C until ready to be complexed with streptavidin and DNA.

### DNA sequence

All the sequences were generated using the program Uniquimer and were purchased from Integrated DNA Technology, Inc (www.idtdna.com). The biotinylated CS strands (Btn-CS) were HPLC purified. The strands S_0_–S_7_ and PolyT were purified by denaturing PAGE. Below are the sequences:

Btn-CS: CATCGAACAATCCGGTCGAGTGCCATGATTTGTGAG/3BioTEG/

PolyT: CTCACAAATCATGGCACTCGACCGGATTGTTCGATGTTTTTTTTTTT

S_0_: CTCACAAATCATGGCACTCGACCGGATTGTTCGATGCGTGACGTAAGTGGT

S_1_: CTCACAAATCATGGCACTCGACCGGATTGTTCGATGACCACTTACGTCACGAAGAG

S_2_: CTCACAAATCATGGCACTCGACCGGATTGTTCGATGCGTAGCTCTTCGTGA

S_3_: CTCACAAATCATGGCACTCGACCGGATTGTTCGATGTCACGAAGAGCTACGATGCT

S_4_: CTCACAAATCATGGCACTCGACCGGATTGTTCGATGGGTTCAGCATCGTAG

S_5_: CTCACAAATCATGGCACTCGACCGGATTGTTCGATGCTACGATGCTGAACCTTCCT

S_6_: CTCACAAATCATGGCACTCGACCGGATTGTTCGATGTTTTTAGGAAGGTTC

S_7_: CTCACAAATCATGGCACTCGACCGGATTGTTCGATGGAACCTTCCTAAAAAGCAAATTTTTTTTTGCTTTTTAG

### Native polyacrylamide gel electrophoresis

The gel demonstrating results of the toehold strand displacement reactions was a 10% native gel. It was prepared in 1 × TAE/Mg^++^ buffer (20 mM Tris-HCl, pH 7.5, 2 mM EDTA, 6.25 mM Mg^++^, pH = 7.6). The DNA structures were annealed following a standard protocol: heating at 95 °C for 5 min followed by cooling to room temperature over ~2.5 h. The gel was run on a Hoefer SE-600 gel electrophoresis unit at 7 V cm^−1^ for 5 h at 12 °C, post-stained with 0.5 μg ml^−1^ ethidium bromide, and visualized by UV transillumination.

### DNA functionalization

Overall, 50 pmol DNA loop structures L_1_, L_2_, and L_3_ (native gel-purified), 100 pmol initiator strand S_0_ and 100 pmol hairpin strand S_7_ were separately annealed with an equimolar amount of biotinylated complementary strands in the magnesium buffer (TMS buffer for the strands functionalized on 4.7 μm droplets; TMT buffer: 10 mM Tris-HCl pH 8, 4 mM MgCl_2_, 1 mM Tergitol TMN-6, 0.1 mM NaN_3_ for the strands functionalized on 3.2 μm droplets, same in the following steps). Then different combinations of fluorescent streptavidin were added to the DNA solutions at a ratio of 1 streptavidin molecule: 1 biotin group. For the streptavidin added to S_0_: 100% were labeled with Alexa® Fluor 647; L_1_: 67% Alexa® Fluor 647 and 33% Alexa® Fluor 488; L_2_: 33% Alexa® Fluor 647 and 67% Alexa® Fluor 488; L_3_: 100% Alexa® Fluor 488; S_7_: 100% Alexa® Fluor 546. Meanwhile, 35 μl (25 μl) of the creamed 4.7 μm (3.4 μm) emulsion droplets were diluted in a 500 μl magnesium buffer, and then split evenly into five parts. After a 30-min incubation, each DNA/streptavidin solution was mixed with one part of the emulsions. The mixtures were incubated for 1 h allowing for the binding of streptavidin to biotin groups on the emulsion droplet’s surface. Then they were washed four times to remove the remaining unbound DNA and excess streptavidin. Each type of droplets were diluted using the same magnesium buffer to a final concentration ~ 3 × 10^3^ μl^−1^.

### Confocal fluorescent microscopy

Leica DM6000 CS confocal microscope (TCS SP5 II) was used to take fluorescent images of our samples. Excitations with 488 nm, 543 nm, and 633 nm were used for imaging the samples. A ×63 oil immersion objective (Leica) was used for all fluorescent imaging.

### Data Availability

The data that support the findings of this study are available from the corresponding author on request.

## Electronic supplementary material

Supplementary Information
