# Supplementary material for: Sequential self-assembly of DNA functionalized droplets
Source: Nat Commun. 2017 Jun 16;8:21. doi: 10.1038/s41467-017-00070-0 (PMC5473892; doi:10.1038/s41467-017-00070-0)
Supplement: Supplementary file 1 — Supplementary Information [file 41467_2017_70_MOESM1_ESM.pdf]

Supplementary Figures

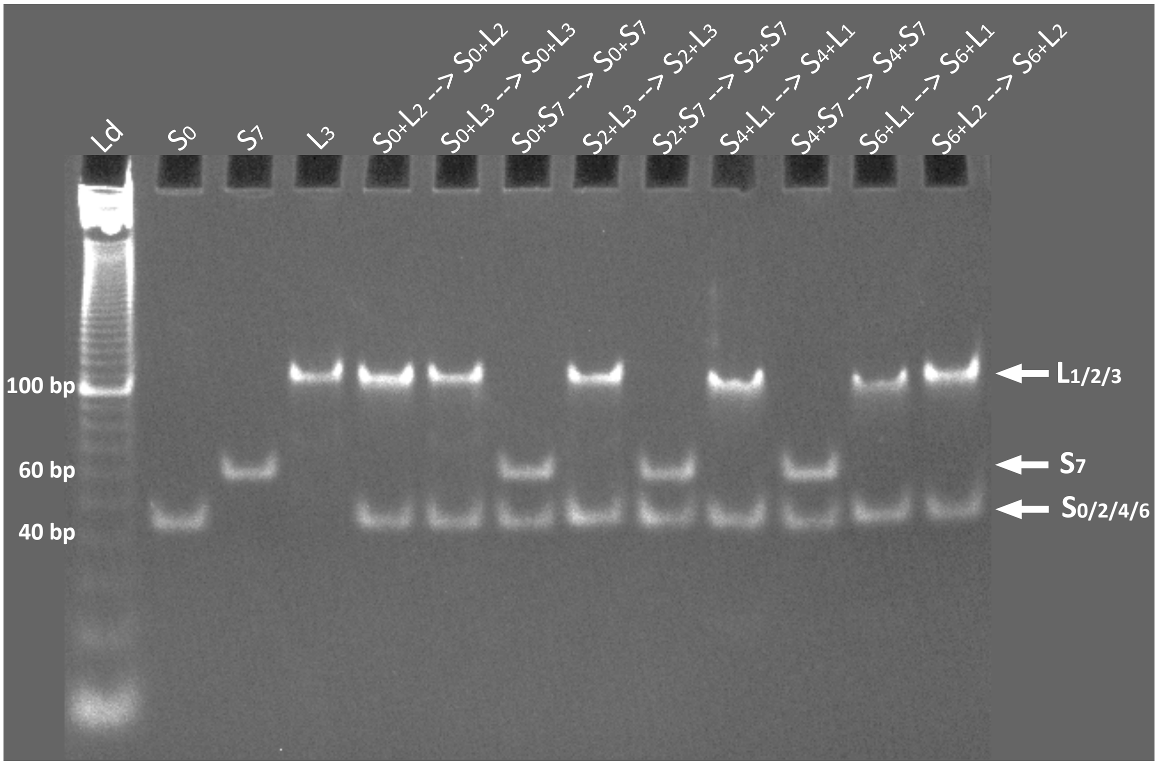

Supplementary Figure 1 | A Native PAGE shows negative controls of other unintended "cross-talk" reactions, leaving the designed sequential assembly the only pathway. Column Ld is the 10 bp ladder. Column S_0 serves as a reference for single strands S_2, S_4, and S_6 with the similar molecular weights. S_7 marks the terminator hairpin. L_3 is a reference for L_1 and L_2 (partially hybridized strands). The results of column S_0 + L_2 to column S_6 + L_2 confirm that all the unintended cross-talk reactions won't happen.

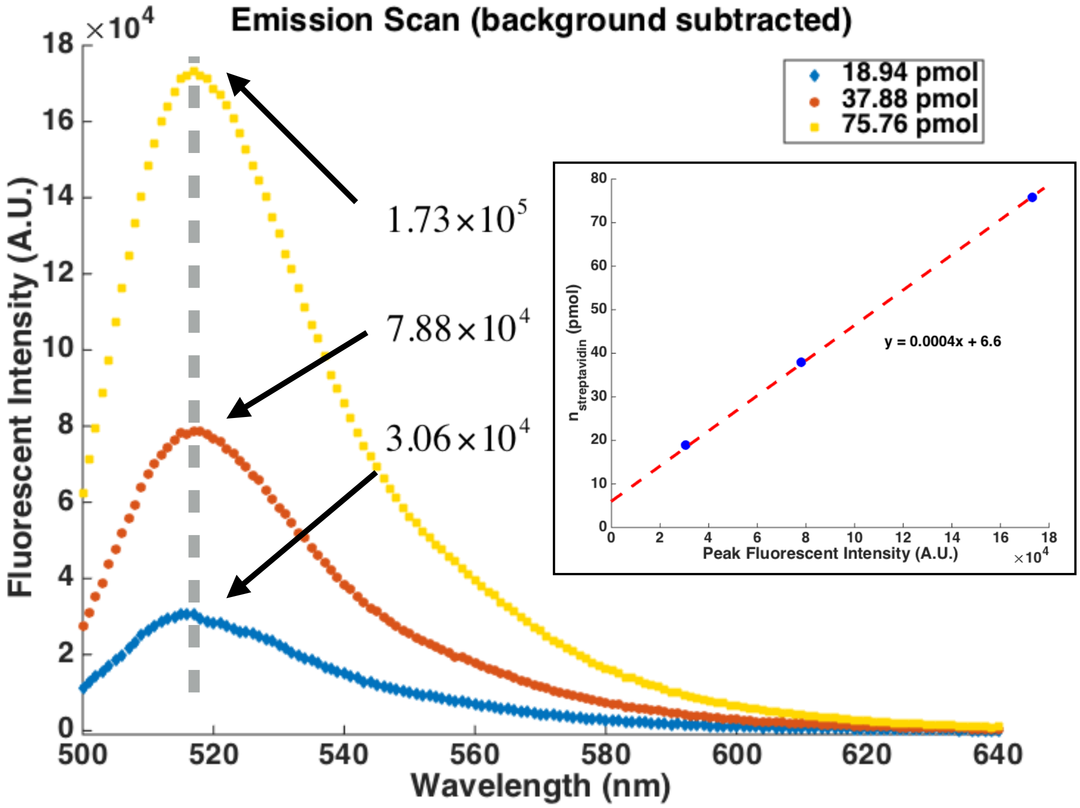

Supplementary Figure 2 | The fluorescence measurements of the subnatants (containing different amounts of unbound fluorescent streptavidin) from different samples. The curve from the control group (sample D) is set as the background (not shown in the figure). The three curves plotted in the figure above are emission fluorescence intensities with the background subtracted. The peak values are labeled and used to calculate the amount of streptavidin bound to emulsion droplets. The inset plots the amount of streptavidin dosed initially versus the peak intensity of the subnatant. We linear fit the data and found the interception of y-axis to be ~6.6 pmol, which is the amount of streptavidin consumed by 5 μl of creamed biotinylated emulsion droplets.

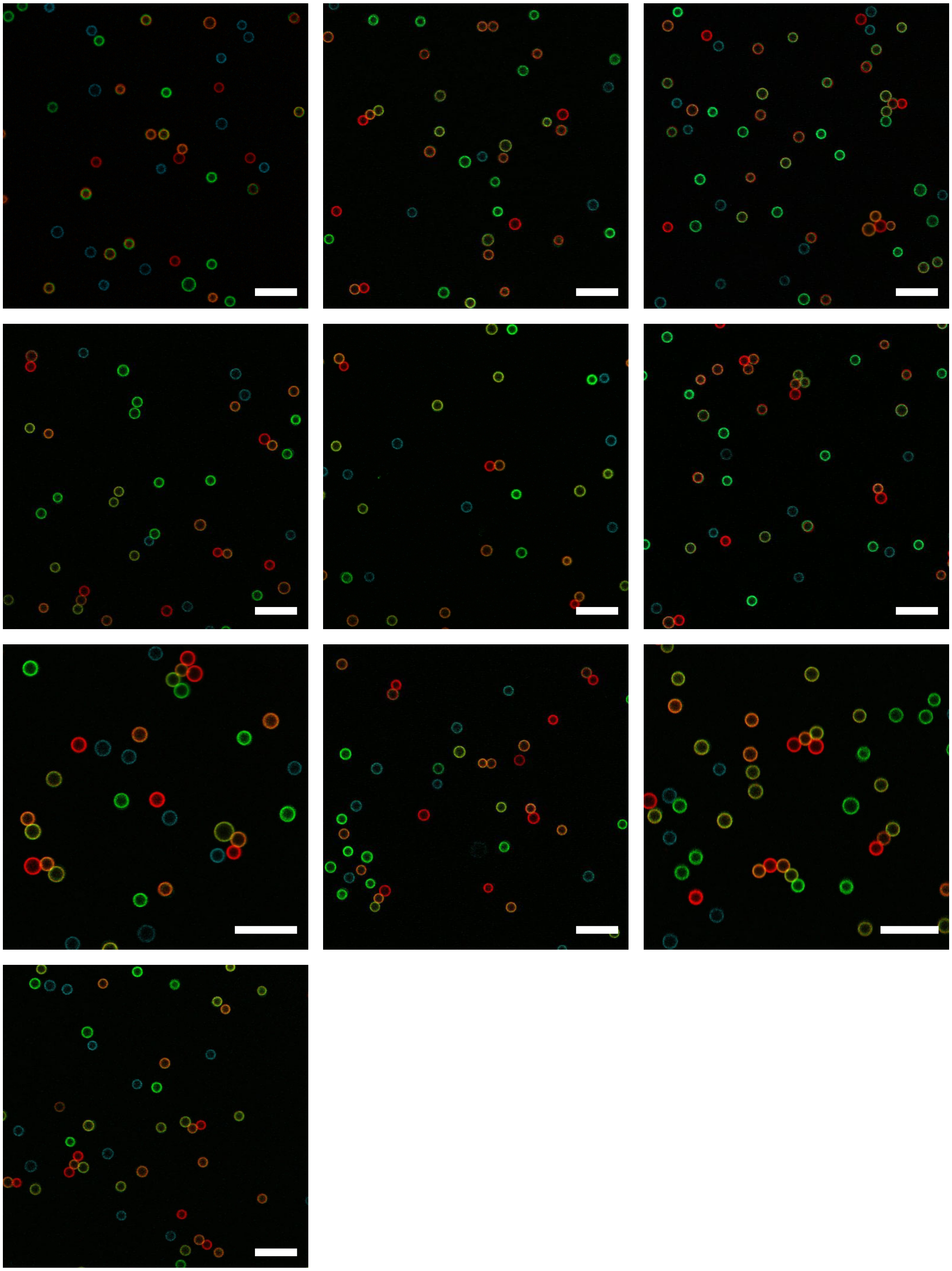

Supplementary Figure 3 | Confocal images taken after 10 hours of incubation for statistical analysis. All scale bars are 20 μm.

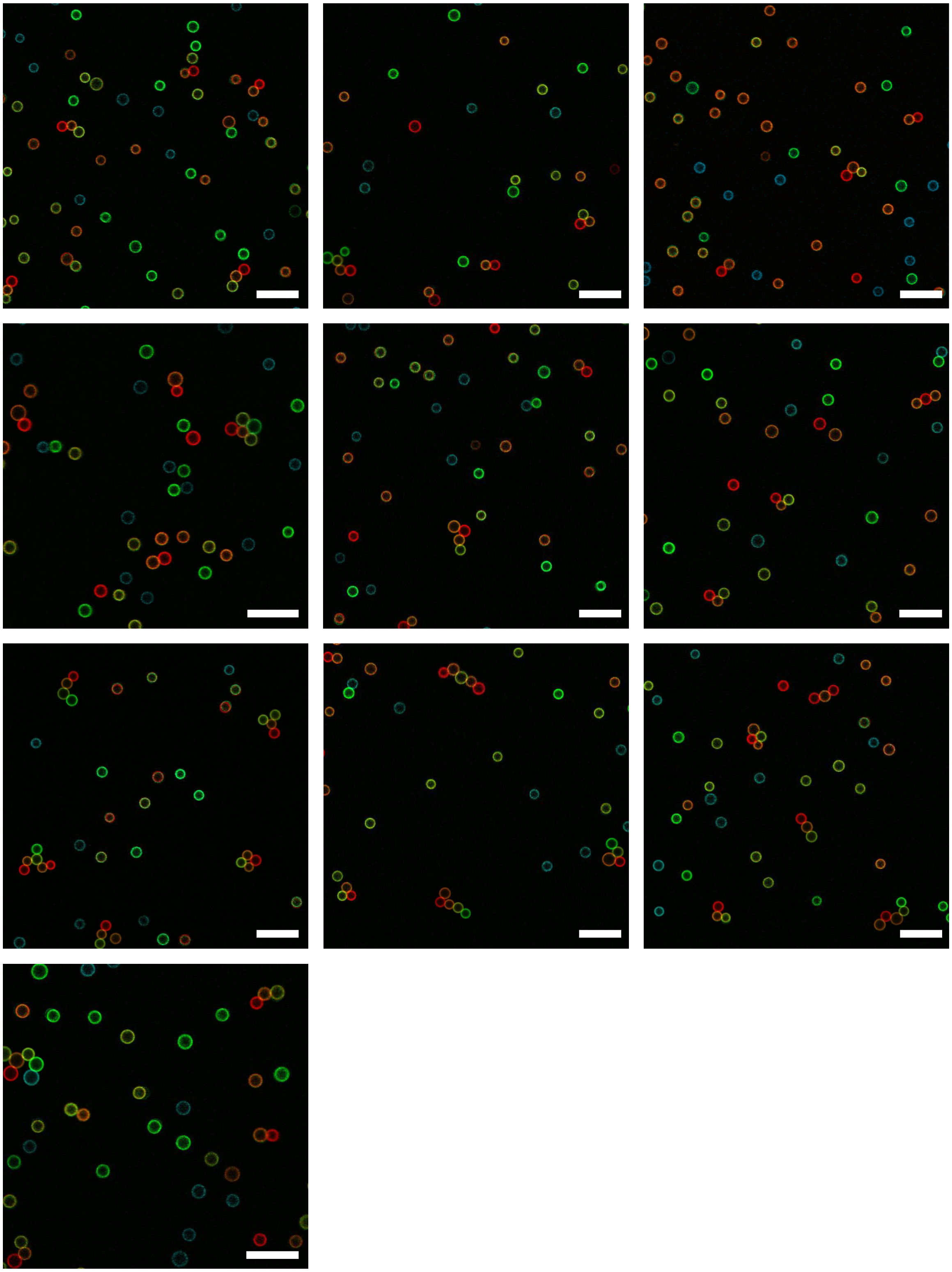

Supplementary Figure 4 | Confocal images taken after 24 hours of incubation for statistical analysis. All scale bars are 20 μm.

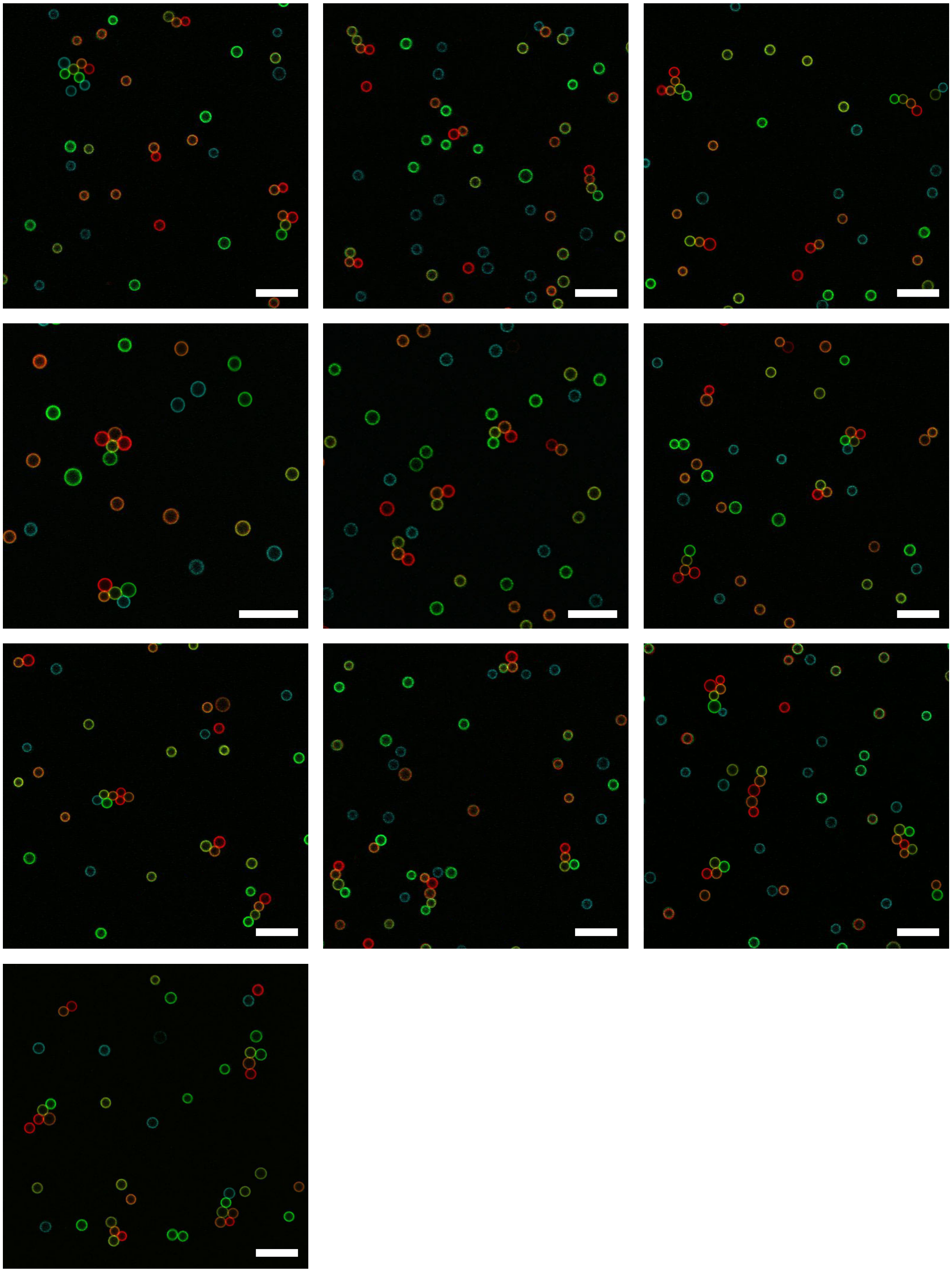

Supplementary Figure 5 | Confocal images taken after 46 hours of incubation for statistical analysis. All scale bars are 20 μm.

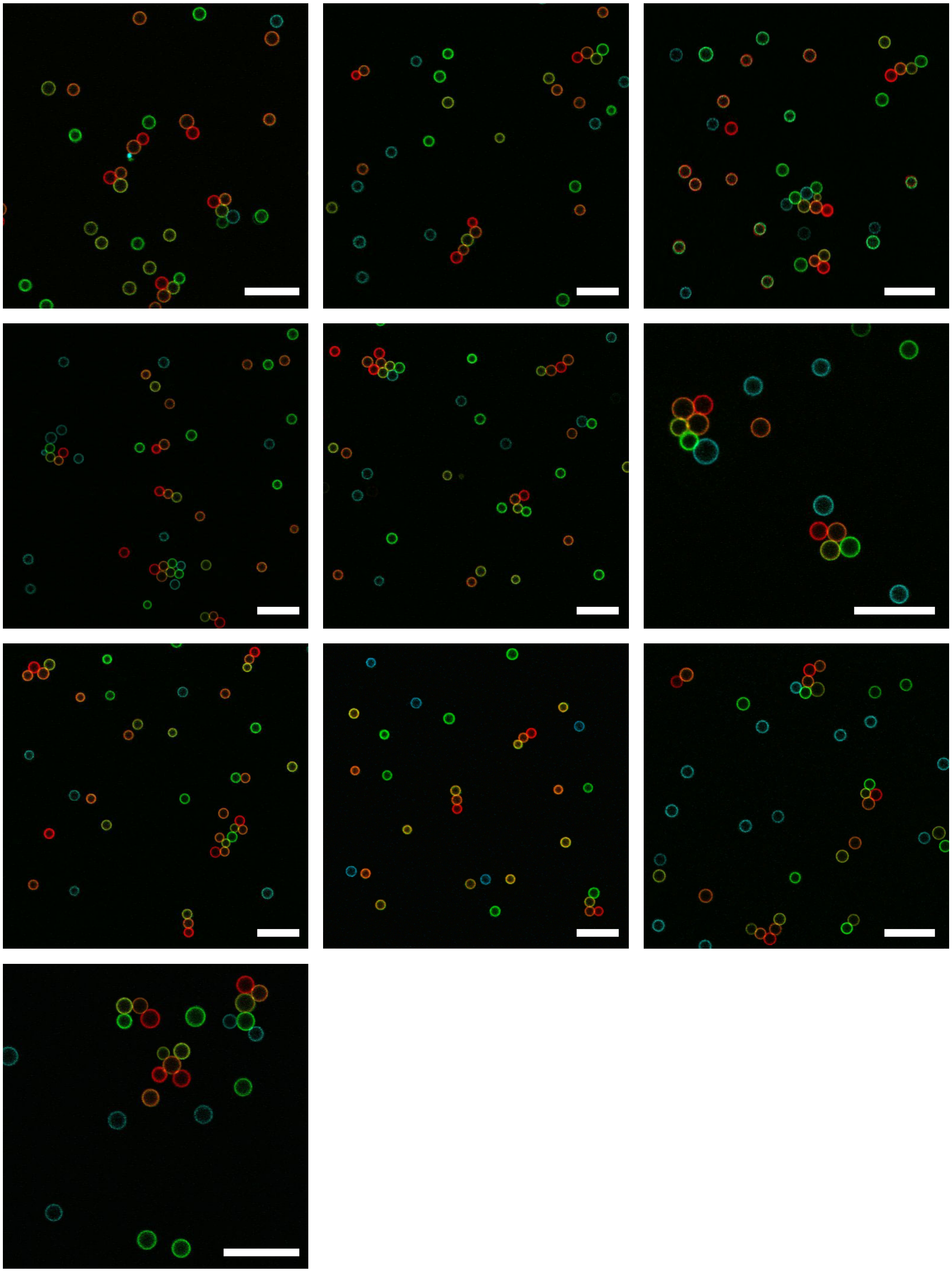

Supplementary Figure 6 | Confocal images taken after 74 hours of incubation for statistical analysis. All scale bars are 20 μm.

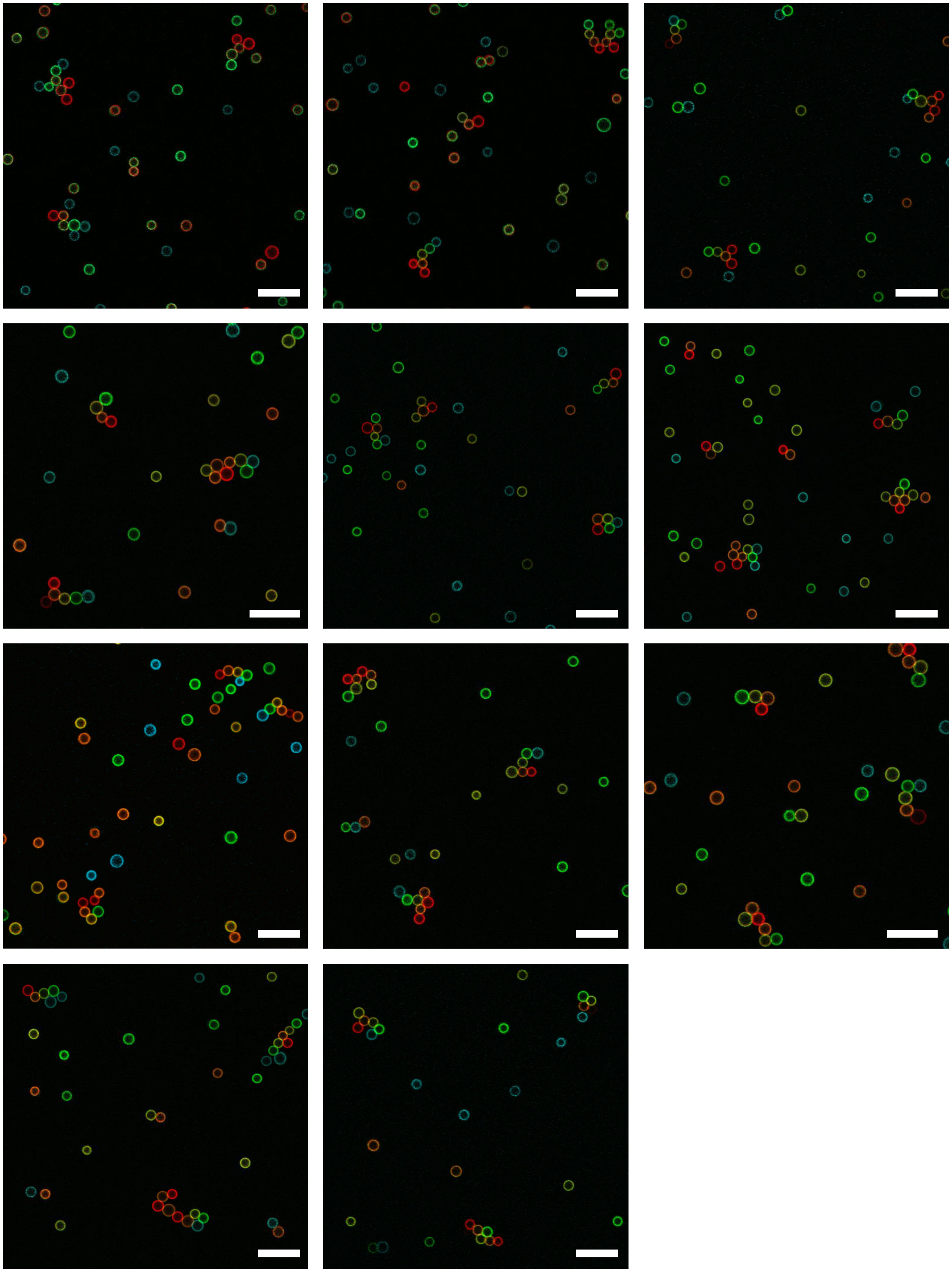

Supplementary Figure 7 | Confocal images taken after 101 hours of incubation for statistical analysis. All scale bars are 20 μm.

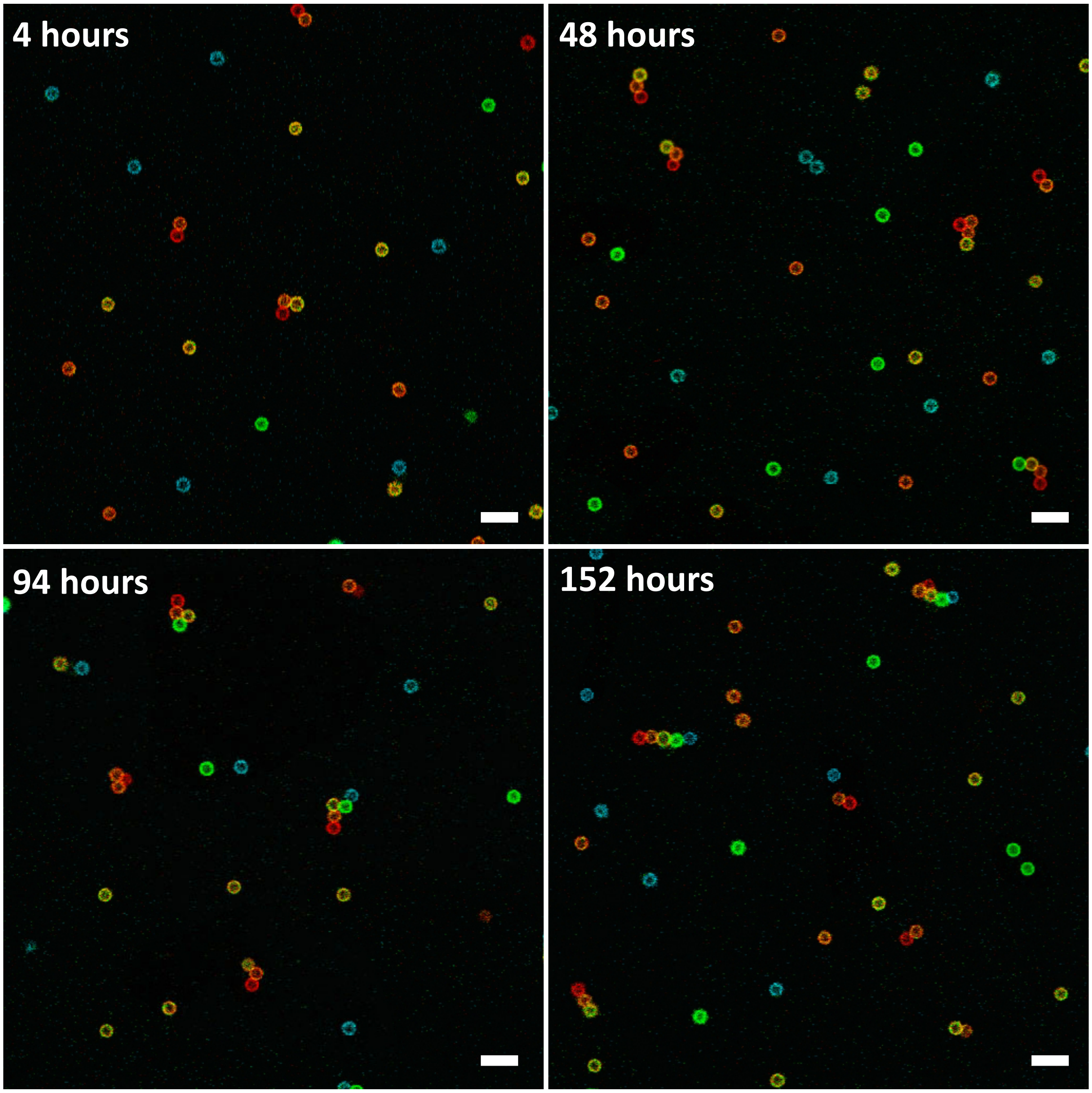

Supplementary Figure 8 | Representative confocal images of the sequential self-assembly using smaller droplets (3.4 µm). The color-coding is the same as in Figure 3. The time scale is a little slower than the one of the 4.7-µm droplets. All scale bars are 10 µm.

Supplementary Tables

Time (hour)	Total number counted	Number of droplets with initiated bindings		Number of droplets with non-specific bindings
		Linear	Branched	
10	544	151	9	6
24	529	194	19	12
46	497	210	31	22
74	476	213	44	30
101	503	239	83	46

Supplementary Table 1 | The numbers of droplets (4.7 μm droplets) with initiated linear/branched bindings and with non-specific bindings.

Time (hour)	Total number counted	In Monomer	In Dimer	In Trimer	In Tetramer	In Pentamer
10	69	23	24.5	16.5	5	0
24	64	11	16.5	20.5	11	5
46	74	10	15	15	23	11
74	59	3	6	11	19	20
101	71	4	8	16	18	25

Supplementary Table 2 | The numbers of initiators (4.7 μm droplets) in various clusters at different time points. '0.5' represents that one initiator branches to two successors.

Time (hour)	Total number counted	Number of droplets with specific bindings		Number of droplets with non-specific bindings
		Linear	Branching	
152	283	107	28	23

Supplementary Table 3 | The numbers of droplets (3.4 μm droplets) with initiated linear/branched bindings and with non-specific bindings.

Time (hour)	Total number counted	In Monomer	In Dimer	In Trimer	In Tetramer	In Pentamer
152	31	1	4.5	6.5	7.5	10.5

Supplementary Table 4 | The numbers of initiators (3.4 μm droplets) in various clusters at different time points. ‘0.5’ represents that one initiator branches to two successors.

Supplementary Methods

Measurement of DNA strands density on droplet. A fixed amount of emulsion droplets (functionalized with biotinylated phospholipids) was mixed with varying amount of streptavidin and then incubated. After centrifugation the subnatants from each sample containing unbound streptavidin were placed in a fluorescent spectrometer (Horiba PTI QuantaMaster 400) for fluorescence measurements. The signal intensities detected from different samples were used to calculate the amount of streptavidin bound to emulsion droplets (Supplementary Fig. 2). We first diluted 20 μl of creamed emulsion (4.7-μm droplets) in 600 μl TMS buffer, and split it into four parts (labeled with sample A, B, C and D) evenly. 2 μl, 1 μl, 0.5 μl and 0 μl (control group) of Alexa® Fluor 488 streptavidin (37.88 μM) were added to sample A, B, C and D respectively. The samples were then gently tumbled at room temperature for 0.5 hr. After incubation, the samples were centrifuged to cream the droplets. From each sample, 5 μl of the subnatant was taken out and diluted to 800 μl with the same buffer for fluorescence measurements. The curve from the control group (sample D) was set as the background. The three curves plotted in the figure above were emission fluorescence intensities with the background subtracted. The peak value of each curve should be proportional to the amount of unbound streptavidin. It can be written as $I_{peak} \propto n_{subnatant} = n_{tot} - n_{emulsion}$, where $n_{subnatant}$, n_{tot} and $n_{emulsion}$ refer to the amount of streptavidin in the subnatant, the amount dosed at the beginning, and the amount bound to emulsion, respectively. Plotting n_{tot} against I_{peak}, we then linear fit the data and found $n_{emulsion}$ from the interception of y-axis to be 6.6 pmol. Knowing that the volume fraction of a random close packing of droplets is ~0.6 and the average radius is 2.35 μm, we can calculate the number of droplets in 5 μl of creamed emulsion. The coverage of streptavidin molecules on the droplet surface is then obtained as ~1000 /μm^2. This is also used as the surface coverage of DNA strands in our paper, assuming 1: 1 binding ratio of streptavidin to biotinylated DNA. For the 3.4-μm droplets we assume they have similar surface coverage because they showed similar brightness from confocal fluorescent microscopy.

Chamber preparation. The flow chamber has a sandwich structure with a glass slide as the bottom layer, two small pieces of glass slides (thickness = 1 mm) as spacers and a cover slip on the top. The dimension of the chamber is 10 mm×2 mm×1 mm (~20 μl volume). The cover slip was plasma etched first and then treated with HMDS (hexamethyldisilazane, from Sigma Aldrich) overnight. Before loading sample, the chamber was soaked in a 1% pluronic F127 buffer for 2 minutes and then gently dried with filtered nitrogen gas. After the sample was loaded, the two open ends were sealed with UV curing adhesives (NOA 68, Norland Products, Inc.).

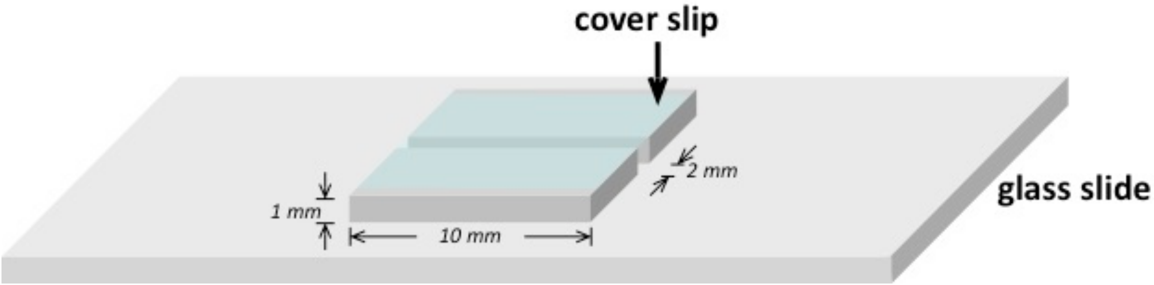

Supplementary Note 1

Kinetics model for the initiated polymerization process.

For 4.7 μm droplets, the average aggregation time for each step can be written as [1]:

$$\tau = \tau_{DLA} + \frac{\tau_R}{4\pi R_d L C_0}\ . \tag{1}$$

τ_{DLA} can be calculated from the equation:

$$\tau_{DLA} = \frac{-\ln(4\pi e R_d^2 C_0) - 1}{8\pi D_0 C_0}\ . \tag{2}$$

The number densities of B, C, D and E are equal, leading to an identical τ_{DLA} for each step of binding. Using R_d = 2.35 μm, $C_0 = 6\times10^2$ mm^{-2} and D_0 = 0.037 μm^2 s^{-1}, we get τ_{DLA} = 35 minutes (~ 0.5 hour) from the above equation.

In the experiment one part of the active DNA strands S_0, along with four parts of neutral strands (Poly-T), are pre-coated onto the surface of droplet A. This 20% ratio is chosen to lower the branching ratio, while not greatly slowing down the binding. The measured streptavidin density on surface (equivalently the overall surface coverage of DNA strands) is ~ 1000/μm^2. The number density of S_0 is ~ 200 μm^{-2}.

In comparison the active strands on droplets B, C or D are released from the adhesion patches formed from the previous binding, and is determined by the size of the patch. A previous study [2] shows that the arrangement of DNA binders within the patch can be approximated as 2D close packing of streptavidin. Thus $N_{DNA} = A_{patch}/A_{stvd}$. From our measurements the average size of an adhesion patch is around 400 nm in diameter, equivalently A_{patch} ~ 0.13 μm^2. Then the surface coverage of active strands on droplet B, C or D is around 20 μm^{-2}. This discrepancy accounts for the difference of τ_R for A – B binding and τ_R for the B – C, C – D and D – E bindings.

In the model [2], τ_R is the reaction time of a pair of droplets with complementary sticky-end DNA on their surfaces staying within the reaction region (thickness L ~ 20 nm; area S ~ 0.1 μm^2). τ_R is reduced by the number of possible bonding configurations N_G from the equation $\tau_R \approx \tau_r/N_G$, where τ_r is the reaction time if there is only one active strand in the reaction region. N_G is the number of active strands in the reaction region, which is the product of the number density of active strand and the area of the reaction region. N_G ~ 20 for A – B binding, and ~ 2 for B – C, C – D and D – E binding. τ_r is estimated as

$$\tau_r \approx \text{translational search time} \times (1 + \text{encounters needed}) \approx \tau_d\,(1 + \frac{\tau_h}{\tau_t}). \tag{3}$$

In our case there is only one active strand on a droplet and many receptor strands (~ 500 strands/μm^2 for DNA loops) on the other. τ_d can be calculated using smoluchowski diffusion equation:

$$\tau_d = \frac{-\ln(4\pi e R_{DNA}^2 C_{DNA}) - 1}{8\pi D_{DNA} C_{DNA}}\ , \tag{4}$$

where C_{DNA} = 500 μm^{-2}, D_{DNA} ~ 1 μm^2 s^{-1} [2] and πR_{DNA}^2 is the area that one strand occupies (~ 20 nm^2). Putting these parameters into the above equation, τ_d is ~ 100 μs. τ_h is the hybridization time for a pair of complementary DNA strands on the opposing droplets

to bind when the droplets are held within an interaction distance, and τ_t is the translational transit time for a pair of complementary DNA strands to stay in their interaction zone. Previous study [1] has shown τ_h is on the order of 1 s to 10 s, and τ_t is approximately 20 μs. With all the numbers plugged in, τ_r is 5 s to 50 s, and τ_R is 0.25 s to 2.5 s for A – B binding, and 2.5 s to 25 s for B – C, C – D and D – E binding. The second term on the right hand side of Equation (1) ranges from 0.2 hours to 2 hours for A – B binding, and 2 hours to 20 hours for B – C, C – D and D – E binding. Then the average aggregation time τ ranges from 1.2 hours to 3 hours for A – B binding, and 3 – 21 hours for B – C, C – D and D – E binding.

We also repeated the experiment using smaller droplets (diameter ~ 3.4 μm) to see if the kinetics of the polymerization process will change. Below is the calculation of the above model using the parameters for the 3.4 μm droplets.

Using R_d = 1.7 μm, C_0 = 12×10^2 mm^{-2} and D_0 = 0.08 μm^2 s^{-1}, we first get τ_{DLA} = 8 minutes from supplementary equation (2). The coverage of the initiator strands S_0 on droplet A is 10%. The average size of the patch shrinks due to the decrease of the droplet size. Here we simply assume at equilibrium the enthalpy gain from DNA hybridization ($\propto$ the number of bonds, which is proportional to A_{patch}) balances the energy burden from the deformation ($\propto(A_{patch}/A_{droplet})^2$). Thus A_{patch} is proportional to the forth order of the droplet's size, which is around 0.04 μm^2 for R_d = 1.7 μm. Consequently, the surface coverage of active strands on droplet B, C or D is around 12 strands/μm^2. Also the numbers of active strands within the reaction region change to N_G ~ 5 for A – B binding; and ~ 0.6 for B – C, C – D and D – E binding. τ_d, τ_h and τ_t are the same as in the previous situation. Thus τ_R ranges from 1 s to 10 s for A – B binding, and 8 s to 80 s for B – C, C – D and D – E binding. Consequently, the second term on the right hand side of Equation (1) ranges from 0.5 hours to 5 hours for A – B binding, and 4 hours to 40 hours for B – C, C – D and D – E binding. Since τ_{DLA} is much less, the second term can be directly used as the average aggregation time in this situation.

From the calculation we can see the kinetics of the assembly is not much changed by using 3.4 μm droplets. This is also confirmed in the experiment, as shown in Supplementary Fig. 3. The reasons are as follows: (1) Smaller droplets with higher diffusion coefficient encounter more frequently (decrease τ_{DLA}); (2) However the area of the patch formed from each binding is much smaller due to its quadratic dependence on the droplet size, which indicates there are much less DNA strands released from each step of reaction. This increases τ_R ,and as a consequence, balances the decrease of τ_{DLA}.

Supplementary References

1. Wu, K.-T. *et al*. Kinetics of DNA-coated sticky particles. *Phys. Rev. E* **88**, 022304 (2013).

2. Feng, L., Pontani, L.-L., Dreyfus, R., Chaikin, P. M. & Brujic, J. Specificity, flexibility and valence of DNA bonds guide emulsion architecture. *Soft Matter* **9**, 9816–9823 (2013).
